# Health impacts of climate change and health and social inequalities in the UK

**DOI:** 10.1186/s12940-017-0328-z

**Published:** 2017-12-05

**Authors:** Jouni Paavola

**Affiliations:** Centre for Climate Change Economics and Policy (CCCEP), School of Earth and Environment, University of Leeds, Leeds, LS2 9JT UK

**Keywords:** Climate change, Climate change impacts, Health outcomes, Health and social inequalities, Vulnerability, Health and social care

## Abstract

This article examines how social and health inequalities shape the health impacts of climate change in the UK, and what the implications are for climate change adaptation and health care provision. The evidence generated by the other articles of the special issue were interpreted using social justice reasoning in light of additional literature, to draw out the key implications of health and social inequalities for health outcomes of climate change. Exposure to heat and cold, air pollution, pollen, food safety risks, disruptions to access to and functioning of health services and facilities, emerging infections and flooding are examined as the key impacts of climate change influencing health outcomes. Age, pre-existing medical conditions and social deprivation are found to be the key (but not only) factors that make people vulnerable and to experience more adverse health outcomes related to climate change impacts. In the future, climate change, aging population and decreasing public spending on health and social care may aggravate inequality of health outcomes related to climate change. Health education and public preparedness measures that take into account differential exposure, sensitivity and adaptive capacity of different groups help address health and social inequalities to do with climate change. Adaptation strategies based on individual preparedness, action and behaviour change may aggravate health and social inequalities due to their selective uptake, unless they are coupled with broad public information campaigns and financial support for undertaking adaptive measures.

**Electronic supplementary material:**

The online version of this article (10.1186/s12940-017-0328-z) contains supplementary material, which is available to authorized users.

## Background

The Working Group II contribution to the Fifth Assessment Report of the Intergovernmental Panel on Climate Change (IPCC) considers that by 2050 climate change will mostly exacerbate existing health problems, and that populations that are currently most affected by climate-related illnesses will also be at the greatest risk in the future [1]. For example, climate change-induced under-nutrition will mainly occur in currently food-insecure areas [1]. In the UK, climate change will directly influence health outcomes through changing exposure to heat and cold, air pollution, pollen, food safety risks, disruptions to access to and functioning of health services and facilities, emerging infections, flooding and other reasons such as water-borne diseases and increased exposure to UV radiation [2–4], as well as indirectly via for example changing prices of and access to food and energy [5].

Health impacts of climate change will not be the same for all because of the differential exposure, sensitivity and adaptive capacity of individuals and groups, which together constitute their vulnerability [6]. For example, people living in flood plains or in top floor flats can be disproportionately exposed to climate change impacts that can affect their health. Older people and people with medical conditions can be disproportionately sensitive to climate change impacts. Finally, isolated people, those with limited mobility and immigrants with limited language skills and local knowledge may have limited adaptive capacity. The nature of health outcomes an individual or a group experiences is determined by the way in which their exposure, sensitivity and adaptive capacity constitute their vulnerability: when they are aligned vulnerability is compounded.

While health and social inequalities have often been examined in the literature on health care and medicine [7, 8], and social justice analysis has formed an established strand of climate change adaptation literature [9, 10], the intersection between these two areas of research has received less attention to date (but see [11]). It does merit further attention, however, because the incidence of the impacts of climate change on health is not equal, and because pre-existing social and health inequalities shape the outcomes that people will experience in changing climate. This article contributes to addressing this gap in the literature.

## Review

The other articles of this special issue have reported findings regarding the future exposure to heat and cold, air pollution, pollen, food safety risks, disruptions to access to and functioning of health services and facilities, emerging infections and flooding as key pathways through which climate change will impact on health. Other pathways through which climate change will impact on health such as water borne-diseases and changed exposure to UV radiation will be omitted here, as they were not examined in the other contributions to this special issue. In what follows, the evidence reported by the other articles is re-interpreted in light of additional literature and social justice reasoning to draw out the key implications of health and social inequalities for the health outcomes of climate change and vice versa.

## Hotter summers and heat waves

Climate change is expected to increase annual mean temperature by 2–5°C and increase the frequency and intensity of heat waves in the UK by 2100. Research has established a link between increased mortality and increased temperature and heat waves [12]. Heat related excess deaths occur primarily as a result of respiratory and cardiovascular illnesses [13]. In England and Wales, mortality increases 2.1% for each 1°C increase in temperature above the 93rd percentile of average yearly temperature. The excess deaths caused by increased temperature can include substantial mortality displacement or “harvesting”, while it plays smaller role in mortality due to heat waves [13]. Heat related mortality is projected to increase in the UK by 45% by 2020s and by 167% by 2050s when accounting for projected population growth and demographic change [12].

Exposure to heat is greater in the South and East of the country, and people living in urban settlements are more exposed than those living in rural areas due to the urban heat island (UHI) effect [14]. Densely built neighbourhoods with limited open space and green areas increase people’s exposure to heat, but the geometry of the buildings and how they are built also influence exposure [15]. Top floor flats experience greater thermal stress than ground floor flats. Ventilation has substantial influence on heat exposure – it may be constrained by physical building design or by considerations for crime, safety, noise and air pollution [16]. Many of these factors leading to greater exposure come together in deprived urban neighbourhoods.

Older people are more sensitive to heat because of their weaker ability to thermo-regulate [15], and because they have other medical conditions. They are also more likely to have prescribed medication, some of which is associated with increased risk for heat related death [13]. Their adaptive capacity may be limited by isolation or lack of information, mobility or autonomy. Lack of autonomy, and lack of care staff awareness and preparedness, may for example prevent or obstruct behavioural and other adaptations in residential or nursing homes [17]. Alignment of the above factors accentuates vulnerability.

## Milder winters

Cold weather causes thousands of excess deaths in the UK annually. Many of them are caused by cardio-vascular and respiratory illnesses, which are aggravated by cold spells [18]. Cold-related excess deaths do not mainly occur in already frail individuals near the end of their lives unlike is the case with heat-related deaths [18]. Milder winters are predicted to reduce winter mortality and morbidity by 9% by 2020s and 26% by 2050s despite population growth and demographic change that will increase the size of vulnerable population [12] (cf [19]).

Fuel poverty and poor housing elevate exposure to cold-related deaths [18]. Older people often suffer from both and can thus be more exposed than other groups. Fuel poverty has also a gender dimension: women form over two thirds of pensioner population and of economically inactive single person households that are particularly likely to suffer from fuel poverty [20]. Those living in council housing may be less exposed than those living in their own property or renting privately, as income limitations may prevent older people from improving thermal comfort of their home. They are also more sensitive to cold because they cannot thermo-regulate their bodies as well as other age groups [18]. Sensitivity to cold is increased by pre-existing medical conditions such as cardiovascular or respiratory diseases, which are more prevalent in older age groups. Limited economic and informational resources in turn may weaken the adaptive capacity of older people.

A warmer climate may thus generate health benefits by reducing cold-related mortality and morbidity among the vulnerable people. But while milder winters can reduce cold-related deaths, their causation is not well understood [21]. Also, demographic change will make a larger number of people sensitive to cold than today [22]. Increasing energy prices may make it more difficult for exposed and sensitive people to adapt by increasing indoor temperature. There is also uncertainty regarding how higher annual and winter average outdoor temperatures will be achieved: they may be consistent with the continued occurrence of cold spells in the future.

## Air pollution

The pollution of air by nitrogen dioxide (NO_2_), ozone (O_3_) and particulate matter (PM) is associated with increased all-cause and cardiovascular mortality and morbidity, and exposure to elevated concentrations of ozone over shorter periods of time is associated with increased respiratory mortality and morbidity [23, 24]. Warmer weather, more frequent heat waves, changes in rainfall and altered volatile organic compound concentrations may increase future O_3_ and PM concentrations [23]. The evidence from recent heat waves such as that of 2003 suggests that in the UK a third of the excess mortality experienced during a heat wave may be caused by exposure to elevated concentrations of O_3_ and PM_10_ [23, 25].

NO_2_ concentrations are particularly high in cities near major transport corridors where socio-economically deprived and poor people and ethnic minorities are over-represented [26, 27]. If PM concentrations have similar distribution patterns then their increase in changing climate would increase the exposure of the same people to the adverse health outcomes of PM. The situation with O_3_ is more complex as high urban NO and N0_2_ concentrations inhibit the formation of O_3_. However, high O_3_ concentrations can prevail in cities in future if public policies do succeed in bringing down urban nitrogen oxide emissions [28].

Social deprivation and age pre-dispose people for cardiovascular illnesses, which in turn compounds the effects of elevated O_3_ and PM concentrations on their health. In 2008, the most deprived quintile in the UK experienced 50% higher cardiovascular disease mortality than the least deprived quintile [8]. Women in routine jobs experience five times higher cardiovascular disease mortality than women in managerial and professional jobs. These differences in cardiovascular mortality risk and sensitivity to O_3_ and PM pollution emerge from differences in the levels of deprivation, lifestyles, health literacy, access to health services, and environmental exposure [7]. Social deprivation and ethnicity can also constrain adaptive capacity by limiting ability to relocate and to take other measures to avoid exposure or to reduce sensitivity.

## Pollen and human health

Pollen is one key factor in asthma, which can trigger inflammation of airways, coughing and breathing difficulties among people whose immune systems have become hypersensitive to triggers like pollen [29]. Birch and related trees are a key source of pollen in the early pollen season, replaced by grasses later in the season: warming will prolong pollen season and can lead to appearance of new sources of pollen when plant species mix changes due to warming. High pollen levels occur during calm and warm high-pressure systems, and rain and westerly winds lower pollen levels because of wet deposition. Events such as thunderstorms can transform pollen into biological aerosols containing allergens and leading to asthma outbreaks. Therefore, global warming and associated increased frequency and intensity of extreme weather events can aggravate the human health impacts of pollen [29].

While exposure to pollen may be greater in rural areas, trees and weeds lead to pollen exposure in urban areas as well. Asthma has nearly 10% prevalence in the UK population, but among 6–7 year olds its prevalence is over 20% [29]. Asthma is more common among lower socio-economic status people and among Afro-Caribbeans but the role of pollen in the incidence of asthma is not well understood [29]. While asthma is more common in the young, a study of asthma mortality in the United States between 2006 and 2008 indicates that older people with asthma have 5 times higher mortality risk from asthma than younger people [30]. Older people also more frequently experienced other adverse outcomes due to acute asthma in the United States [30]. Other medical conditions such as cardiovascular disease are common co-morbidities of asthma in older people [31].

## Food borne diseases

Prevalence and impacts of food safety related pathogens campylobacter and salmonella is likely to be affected by climate change. Campylobacter cases have increased and in 2006 they led to 18,000 hospitalisations, 80 deaths and economic losses of £600 million per annum in England and Wales [32]. However, the projections for future campylobacter cases are uncertain because different drivers influence cases occurring via dietary and environmental pathways. Salmonella cases have decreased: 9000–10,000 salmonella cases have been reported to the Health Protection Agency and its successor Public Health England in recent years. These have led to 8500 hospital admissions, 199 deaths and economic losses of £39 million per annum in England and Wales [32]. Climate change may countervail the reduction in salmonella cases brought about by improved food hygiene over the past two decades.

In terms of exposure, campylobacter is more prevalent in rural areas and in areas with less social deprivation [32, 33]. It is also more common in infants than in adults and older age groups. Poultry is the most common dietary source of campylobacter. The geographic prevalence of salmonella has not been studied in the UK. Salmonella is typically contracted from raw or undercooked eggs or poultry and it is most common in small children. The elderly and households with small children are the two groups for whom food safety will be a particular problem because of the greater sensitivity of the infants and elderly. Their situation can be exacerbated by low incomes, isolation and other factors that reduce adaptive capacity.

## Health and social care systems

Climate change will affect future health outcomes directly through extreme weather events such as heat waves, cold spells, and flooding impacting on the built infrastructure and social and institutional systems of health care provision, and indirectly due to induced changes in the volume and structure of demand for health care [34]. Heat waves and cold spells can put systems of health care under pressure because of the mortality and morbidity increase for cardiovascular and respiratory diseases and associated increases in hospitalisations [34]. Floods in turn result in increased accidents and emergency visits. Climatic extremes can increase ambulance call out rates by 25–35%, which is comparable to increases related to major flu epidemics [35]. They also increase mental health problems and demand for services addressing them, and influence the length of time that support addressing them is needed [35]. Direct impacts of extreme weather on health care systems include heat stress on inpatients and adverse health outcomes associated with heat stress discussed above, potential care and service disruptions because of power outages, delays in emergency responses and reduced access to health care because of the impacts of flooding and extreme weather on transport infrastructure and services, and reduced staffing and capacity for the same reasons in health care provision [34].

Inpatients and people with urgent medical needs will be most exposed to the impacts of extreme weather on health care systems. Rural dwellers are more exposed to disturbance from cold spells and flooding, while urban residents are more exposed to disturbance due to heat waves. Many of the exposed will be older people who are sensitive to care disruptions and reduced access to care because of pre-existing medical conditions such as cardio-vascular disease and respiratory diseases, which are aggravated by climate change impacts. Adaptive capacity is restricted among those who are in residential care and have limited control over their circumstances, and among those who are isolated or have reduced mobility.

## Emerging infections

Emerging infections are newly appearing in a population or rapidly increasing in incidence or expanding in range, for reasons of which climate change is only one [36]. Climate change can lead to emerging infections when it influences pathogens causing a disease: this is most likely when pathogens spend part of their lives outside the host, making vector-borne diseases the most important infections influenced by climate change [37]. For example, climate change is expected to influence the distribution of malaria, West Nile virus, Chikungunya fever, dengue, Leishmaniasis, Lyme’s disease and tick-borne encephalitis (TBE). While climate change could lead to the introduction of more exotic vector-borne diseases such as Chikungunya fever, West Nile virus and the variant of Malaria caused by *Plasmodium Vivax* over the longer term, the changed occurrence of Lyme’s disease and TBE are the more immediate health risks posed by climate change [38].

Health and social inequalities influence the outcomes of emerging infections in many ways. Factors influencing and patterns of vector ranges can lead to North-South, urban-rural and other gradients in exposure. Differential exposure can align with income or/and age disparities associated with differences in sensitivities and adaptive capacities. For example, if older rural population is more exposed Lyme’s disease and TBE, the people at greater risk of these diseases would also be more likely to have other pre-existing medical conditions and thus likely to experience more adverse health outcomes because of the emerging infections. People in rural areas may have lower adaptive capacity because of more limited access to health care services and medical expertise on emerging infections.

## Flooding

Climate change is likely to increase the frequency and intensity of surface, riverine and coastal flooding in the UK [39]. Every sixth property (5.2 million properties) is exposed to some flood risk in the UK [40]. Figures for the number of people exposed to flooding in the UK range from 1.5 million to 5 million [41, 42]. Damage caused by flooding to buildings and contents of residential and non-residential properties amounts to about £1.3 billion per annum on average and in years of severe flooding such as 2007 losses can double. The above figures exclude loss of income due to disturbance caused by flooding and additional expenses to make alternative arrangements for leisure, work or business. These indirect losses can add 25–50% to direct losses to buildings and contents [42, 43].

Direct health impacts of flooding include drowning, electrocution and other accidental deaths and injuries. Indirect health impacts can occur due to contamination and loss of water supply and loss of access to transport, electricity supply and communications [44–46]. Economic losses and disturbance can also lead to mental health problems [47]: people flooded by the 2007 summer floods in Gloucestershire and Yorkshire were 2–3 times more likely than non-affected people to report mental health problems [48–50]. Flooding can also undermine health care provision by overwhelming the capacity of emergency services, causing power cuts and supply disruptions, and flooding health care facilities [46, 51].

The risk of flooding and its adverse health impacts are unevenly distributed in several ways. Those living in affordable housing and socio-economically disadvantaged households are over-represented in areas at risk from coastal flooding [52]. While these households are also exposed to riverine flooding, affluent households are particularly exposed to riverine flooding [52]. Second, people with disabilities, chronic illness, young children or dependent on public transport can be more sensitive to flood risk. There is also evidence of gender differences in health outcomes related to exposure to flooding [53]. Finally, the capacity of some groups to adapt or recover can be lower because of low incomes, lack of insurance or other reasons: uninsured losses to assets cannot be recovered and repeated exposure to flooding can deplete vulnerable households’ assets, adversely affect their health and make them more vulnerable in the future [50, 54]. People’s tenure status and type of dwelling may also influence the outcomes they experience.

## Discussion

The previous section examined how climate change is likely to have differential health impacts on different groups of people, because of differences in their exposure, sensitivity and adaptive capacity. Old age, having pre-existing medical conditions and social deprivation are key attributes that make some people more vulnerable than others to the health impacts of climate change and to experience more adverse health outcomes than others. However, other attributes such as gender, living in rural or urban locations, isolation, marginalisation and weak community cohesion can also make people more vulnerable.

Climate change, aging population and public spending cuts on health and social care can combine to increase health and social inequalities related to health impacts of climate change in the future. The number of people who are over 60 years old will increase by 50% by 2035 compared to 2010 [55]. The proportion of people who are over 75 will double by 2060 compared to 2010 [56]. This means that many more people will have pre-existing medical conditions that make them vulnerable to health impacts of climate change when the impacts of changing climate accentuate. The Lancet Commission estimates that demographic change accounts for three quarters of the increase in the number of people exposed to heat waves and that climatic change only accounts for one quarter of them [21]. At the same time, the systems of health care depend on critical infrastructure for the supply of electricity and water and for communication – this critical infrastructure is exposed and sensitive to the impacts of climate change, which can undermine care [51].

But ageing will also – together with income increase, technological change and increase in the cost of health care provision – create pressure to increase spending on health care from the current about 8% of GDP to 12–14% of GDP by 2040 [56]. This can be addressed by increasing public spending, reallocation of public spending from other areas to health care, or rationing or patient co-funding of health care provision. If health care is rationed or co-funded, those with greater needs and limited means will suffer reduced access to health care, which will accentuate health and social inequalities.

Many strategies can address health impacts of climate change and the implications of health and social inequalities for them. These include for example new food safety and building regulations, incentivising the refurbishment of old building stock and urban neighbourhoods, improvements to the operations and infrastructure of emergency services and health care provision, improved advance warning and preparedness systems, development and deployment of new diagnostic and therapeutic solutions and public health campaigns and health education. None of these solutions will suffice on its own because compound inequalities underpin vulnerability [57]: a combination of measures will be needed.

Individuals and organisations have capacity for autonomous adaptation and one strategy is to harness it. The strategy of private preparedness and responsibility would emphasise public information campaigns and public health education to make people aware of the health risks associated with climate change, factors contributing to people’s exposure and sensitivity, and alternatives for avoiding and mitigating adverse health outcomes, so that they can protect themselves. Solutions such as advance warning systems would also support such strategies, signalling that people should deploy adaptation measures.

But strategies relying on people’s own initiative can increase rather than decrease health and social inequalities. Income and wealth inequalities often expose low-income groups to greater risks because of their residential and other choices, and their adaptive capacity can also be lower than that of higher-income groups. For example, income and wealth inequalities and different levels of trust and engagement translate to different uptake of adaptation measures among different groups of people. Educational status, immigration status and age may also influence people’s ability to translate health education into plans and actions, as well as to undertake adaptation measures. There is also evidence that people may not consider taking action to adapt to climate change to be their responsibility [58, 59].

Public preparedness measures on the other hand have good potential for alleviating health impacts of climate change and related health and social inequalities. For example, early warnings for emergency services and public service delivery organisations may confer important benefits to vulnerable groups if they lead to improved preparedness and existence of joined-up contingency and emergency plans in different service delivery organisations [54]. This is clearly the aim of the Heat Wave Plan for England [60]. The emergency services are governed by different legislation and priorities than for example flood risk management, which can make them better able to prioritising vulnerable groups [10, 61].

## Conclusions

Climate change will exacerbate existing health problems in populations that are already most affected and will reduce their resilience to future threats to their wellbeing. Hotter summers, heat waves, milder winters, pollen, air pollution, flooding, emerging infections and food safety are key climate change-related factors influencing health outcomes in the UK. But climate change can also directly impact systems and facilities of health and social care and have indirect impacts on health via changing prices of food and energy.

Climate change will have differential health impacts on different groups of people, because of differences in their exposure, sensitivity and adaptive capacity. Age, pre-existing medical conditions and social deprivation are the key factors that make people vulnerable to health impacts of climate change and to experience more adverse health outcomes than others. But gender, living in rural or urban places, isolation, marginalisation and social embeddedness in and cohesiveness of communities also influence vulnerability. Climate change, aging population, decreasing public spending on health and social care can combine to increase in the future health and social inequalities to do with climate variability and change.

While existing research on climate change impacts and health sheds some light on the implications of health and social inequalities for health outcomes of climate change impacts, less is known about the implications of adaptation responses to address them. Different strategies for avoiding and mitigating health impacts of climate change can have quite different implications for health and social inequalities. For example, health education and public preparedness measures that take into account differential exposure, sensitivity and adaptive capacity of different groups may help to address health and social inequalities. Strategies based on individual preparedness, action and behaviour change may in turn aggravate them due to selective uptake of measures and the lack of engagement of some social groups, unless coupled with broad public information campaigns. More research is needed on the implications of climate change adaptation strategies and measures.

## Additional file

Additional file 1: Open peer review. (PDF 356 kb)
