# Supplementary material for: Health impacts of climate change and health and social inequalities in the UK
Source: Environ Health. 2017 Dec 5;16(Suppl 1):113. doi: 10.1186/s12940-017-0328-z (PMC5773866; doi:10.1186/s12940-017-0328-z)
Supplement: Additional file 1: — Open peer review. (PDF 356 kb) [file 12940_2017_328_MOESM1_ESM.pdf]

Reviewer reports

Title: Health impacts of climate change and health and social inequalities in the UK

Reviewer 1: Sotiris Vardoulakis

This is an interesting and well written paper mainly focusing on the implications of climate change for health and social inequalities in the UK. In this context, the manuscript briefly discusses the key health impacts of climate change in the UK, including temperature effects, air pollution and pollen, food borne diseases, emerging infections, and flooding. Other potential effects of climate change on health, e.g. related to water borne diseases or UV radiation exposure, are not discussed. Although this may be justified, the author should explain on what basis certain risks have been included or excluded from the analysis. I would suggest making reference to the 1st Climate Change Risk Assessment report for the Health Sector (2012), HPA's report on the Health Effects of Climate Change in the UK (2012), and the more recent science evidence report of the 2nd Climate Change Risk Assessment (2016).

Minor revisions

I would suggest mentioning the "UK" in the title – health impacts and inequalities may be different in other countries, particularly in low income settings

Page 4, 2nd paragraph, first line: *"Climate change is expected to increase temperature by 2-5°C".* It is not clear if the author refers to annual mean temperature.

Page 4, 2nd paragraph: *"The excess deaths caused by increased temperature can include substantial mortality displacement or "harvesting",* while it plays a much smaller role in mortality due to heat waves". Please provide a reference for this statement. Harvesting may occur to some extent during heatwaves.

Page 4, 2nd paragraph: *"Heat related mortality is projected to increase in the UK by 45 % by 2020s and by 167% by 2050s"*. Please clarify that this also accounts for demographic changes.

Page 4, 3rd paragraph: *"people living in urban settlements are more exposed than those living in rural areas due to the urban heat island (UHI) effect".* Please provide a reference, e.g. Heaviside et al. 2016. Attribution of mortality to the Urban Heat Island during heatwaves in the West Midlands, UK. Environmental Health 15(1): 49-59.

Page 4, 3rd paragraph*: "Top floor flats experience great thermal stress while ground floor flats do not".* I presume ground floor flat also experience some degree of thermal stress on very hot days.

Page 4, 3rd paragraph: *"Ventilation has substantial influence on heat exposure – it may be constrained by physical building design or by crime and safety considerations".* Additional constraints may be posed by noise and air pollution. Please see: Vardoulakis et al. 2015. Impact of climate change on the domestic indoor environment and associated health risks in the UK. Environment International 85, 299-313.

Page 5, 1st paragraph: *"They are also more likely to have prescribed medication, some of which is associated with increased risk for heat related death"*. Please provide a reference.

Page 5, 2nd paragraph: *"Milder winters are predicted to reduce winter mortality and morbidity by 9% by 2020s and 26% by 2050s"*. Again please clarify that this takes into account demographic changes.

Page 5, 2nd paragraph: *"A warmer climate may thus generate health benefits by reducing mortality and morbidity among the vulnerable"*. This is true for cold-related mortality and morbidity, but it may not be the case for mortality and morbidity in general. I would suggest making this point more specific.

Page 6, 1st paragraph: *"There is also uncertainty regarding how higher annual and winter average temperatures will be achieved"*. It is not entirely clear if the author refers to outdoor or indoor temperature.

Page 6, 2nd paragraph: *"The pollution of air by nitrogen dioxide (NO2), ozone (O3) and particulate matter (PM) is associated with increased all-cause and cardiovascular mortality and morbidity and exposure to ozone is associated with increased respiratory mortality and morbidity".* NO2 and PM have also been associated with increased respiratory mortality and morbidity. Short-term exposure to O3 has been associated with increased all-cause mortality, and respiratory and cardiovascular hospital admissions. Please see COMEAP report: quantification of mortality and hospital admissions associated with ground-level ozone (2015).

Page 6, 2nd paragraph: *"Warmer weather, more frequent heat waves, changes in rainfall and altered volatile organic compound concentrations may increase future O3 and PM concentrations, although their or their pre-cursors' emissions could decrease over time".* O3 is not directly emitted into the atmosphere – it is only a product of atmospheric reactions.

Page 6, 2nd paragraph: *"The evidence from recent heat waves such as that of 2003 suggests that in the UK a third of the excess mortality experienced during a heat wave may be caused by exposure to elevated concentrations of O3 and PM10".* The original reference for this is: Stedman, 2004. The predicted number of air pollution related deaths in the UK during the August 2003 heatwave. Atmospheric *Environment 38 (2004) 1087-1090.*

Page 6, 3rd paragraph: "CO and NO2 concentrations are particularly high in cities near major transport corridors..." Outdoor CO concentrations are generally very low in the UK.

Page 6, 3rd paragraph: *"NOX concentrations"*. Please provide a definition for NOX.

Page 6, 3rd paragraph: *"high O3 concentrations can prevail in future if public policies will bring down NOX emissions"*. I would suggest adding "in cities" – the overall picture is more complex.

Page 7, 1st paragraph: *"differences in cardiovascular mortality risk and sensitivity to O3 and PM pollution emerge from differences in the levels of deprivation, lifestyles, health literacy..."* I am not entirely convinced that deprivation, lifestyle, etc. affect the sensitivity to air pollution. Lifestyle, for example, affects cardiovascular mortality, which compounds the effect of air pollution on this health endpoint.

Page 7, 3rd paragraph: *"While the young are more exposed to asthma"*. I would suggest "…are more affected by asthma"

Page 7, 3rd paragraph: *"older people with asthma have 5 times higher overall mortality risk than younger people"*. It is difficult to interpret this statement. Older people overall have higher mortality risk than younger people. How does asthma affect this relationship?

Page 8, 2nd paragraph: *"Prevalence and impacts of food safety related pathogens campylobacter and salmonella will be affected by climate change"*. This statement conveys too much certainty in my opinion. The rest of the paragraph indicates that there are significant uncertainties.

Page 8, 2nd paragraph*: "…have been reported to the Health Protection Agency in recent years"*. The Health Protections Agency transferred its function to Public Health England in April 2013.

Page 10, 2nd paragraph: *"Emerging infections"*. This section gives the impression that a list of vector-borne diseases (VBD) are likely to appear in the UK due to climate change. It is important to clarify whether the occurrence of these VBD in the UK is plausible under climate change scenarios, and substantiate this with references. For example, please see: Medlock and Leach, 2015. Impact of climate change on vector-borne disease risk in the UK. The Lancet Infectious Diseases 15, 721-730.

Page 10, 2nd paragraph: "climate change is expected to influence the distribution of malaria, West Nile virus, Chikungunya fever, dengue, Leishmaniasis, Lyme's disease and tick-borne encephalitis (TBE)". The geographical context here needs to be made clear. Are these vector-borne diseases likely to become a public health issue for the UK under climate change?

Page 14, 1st paragraph: "avoidance measures". I would suggest "adaptation measures"

Page 15, 2nd paragraph: "More research is needed on the implications of response strategies". Please elaborate. What kind of research is needed? By "response strategies" does the author mean climate change adaptation strategies?

Competing interests declaration: I declare that I have no competing interests.

Reviewer 2: Melani Boeckmann

Summary and proposed revisions

This article presents a summary of the evidence for health effects of climate change in the UK, mediated by social inequalities. The discussion section additionally proposes public spending as a factor mediating ability to adapt to climate change.

Overall, the article covers the main climate change impacts documented and/or expected for the UK as well as their likely health outcomes, including both direct and indirect effects. Social vulnerability links are explored, albeit frequently without citing references for assertions, and without explicitly mentioning gender as a category that is linked to other social inequalities such as income, safety issues in certain spaces etc.

The article does not clearly differentiate between findings of the original articles it aims to summarize and the added literature on climate change and social issues. I believe the article will profit from a different kind of structure, for example, by summarizing each of the articles before combining their findings' links to social justice in a separate section. Alternatively, at least clearly marking where the summary ends and the author's additional findings begin would be helpful to readers. This way the issue of public expenditure and adaptation's potential to increase inequalities would possibly become more visible as a separate section rather than being subsumed under the discussion. I assume that the economic perspective is supposed to be the added value of the article, yet right now the reader finds it hidden towards the end in the discussion section.

In summary, I recommend a major restructuring or rewriting to mark summary and original sections, and minor revision mainly in form of critically assessing where assertions need to be supported by references and to add these. It also not quite clear to me where social justice plays a role in this article: while it discusses inequalities, only in the conclusions does the article state how these are a problem and what adaptation can do to acknowledge and reduce these.

Recommendation

As I did not see the other articles of the special issue for this review, I am unable to say whether the article adequately summarizes their contents.

If the purpose of the article is to summarize previous knowledge without adding much of its own in terms of analysis, then I recommend one major revision (outlined above) and a few minor revisions before acceptance.

If the purpose of the article is to contribute new knowledge to the field, then in its current state it is unable to do so and requires major revisions to form and content. I refer to the journal editor for this decision as they have seen the entire special issue and the cover letter outlining the article's scope.

Major revisions

Please consider revising the article in a way that clearly distinguishes your summary of other articles in this issue from your own additional literature search and social research.

Also consider being more specific about the justice component linked to social inequalities, and about your economic perspective. Does it make sense to keep this in the discussion section, or should it be an additional section in the body of the article?

Please consider specifying links between income, fuel poverty, older people living on their own etc to not only ethnicity and age but also gender. I believe that leaving it out is an unnecessary omission even for high income contexts (see, for example: Adamson, Ben-Shlomo, Chaturvedi, & Donovan, 2003; Lowe, Ebi, & Forsberg, 2013).

Minor revisions

Needs more citations for specific assertions: many statements are made without references, or without clearly linking a previous reference to that statement. See, for example, the sections on older people's comorbidities, salmonella in children, disruptions of health care for rural populations on page 9, social aspects of infections on page 10.

Abstract: Under Methods, the sentence needs to begin with a capital letter.

p.14: please explain why warning systems could reduce unequal preparedness in light of the previous paragraph, where you correctly state that uptake is mediated by social factors. Warning systems also serve to encourage people to protect themselves: how do they differ from other information campaigns?

References:

Adamson, J., Ben-Shlomo, Y., Chaturvedi, N., & Donovan, J. (2003). Ethnicity, socio-economic position and gender--do they affect reported health-care seeking behaviour? *Social Science & Medicine (1982)*, *57*(5), 895–904. Retrieved from http://www.ncbi.nlm.nih.gov/pubmed/12850114

Lowe, D., Ebi, K. L., & Forsberg, B. (2013). Factors increasing vulnerability to health effects before, during and after floods. *International Journal of Environmental*

Research and Public Health, *10*(12), 7015–67. http://doi.org/10.3390/ijerph10127015

Competing interests declaration: I declare that I have no competing interests.

Reviewer 3: Gordon Walker

The paper provides a careful, systematic and generally well-judged summary review of the relationships between the health impacts of climate change and social and health inequalities and vice versa. There are a few places (in the attached file) where I have questioned the summary comments that are made and/or suggested some more nuanced phrasing, and also where I have suggested some additional sources. In general it would be good to have a bit more indication of where evidence, across the field examined, is robust and what it is lacking or rather thin. I have commented on this in one section in particular, but this point more generally applies across the paper.

Competing interests declaration: I declare that I have no competing interests.

Health Impacts of Climate Change and Health and Social Inequalities

Professor Jouni Paavola

Centre for Climate Change Economics and Policy (CCCEP)

School of Earth and Environment

University of Leeds

Leeds LS2 9JT, UK

j.paavola@leeds.ac.uk

Health Impacts of Climate Change and Health and Social Inequalities

Abstract

Background: This article examines how social and health inequalities shape the health impacts of climate change in the UK, and what the implications are for climate change adaptation and health care provision.

Methods: the evidence generated by the other articles of the special issue were interpreted using social justice reasoning in light of additional literature to draw out the key implications of health and social inequalities for health outcomes of climate change.

Results: Exposure to heat and cold, air pollution, pollen, food safety risks, disruptions to access to and functioning of health services and facilities, emerging infections and flooding are examined as the key impacts of climate change influencing health outcomes. Age, pre-existing medical conditions and social deprivation are found to be the key factors that make people vulnerable and to experience more adverse health outcomes related to climate change impacts. In the future, climate change, aging population and decreasing public spending on health and social care may aggravate inequality of health outcomes related to climate change.

Conclusions: Health education and public preparedness measures that take into account differential exposure, sensitivity and adaptive capacity of different groups help address health and social inequalities to do with climate change. Adaptation strategies based on individual preparedness, action and behaviour change may aggravate health and social inequalities due to their selective uptake, unless they are coupled with broad public information campaigns and financial support for undertaking adaptive measures.

Keywords

Climate change, climate change impacts, health outcomes, health and social inequalities, vulnerability, health and social care

Introduction

The Working Group II contribution to the Fifth Assessment Report of the Intergovernmental Panel on Climate Change (IPCC) considers that by 2050 climate change will mostly exacerbate existing health problems, and that populations that are currently most affected by climate-related illnesses will also be at the greatest risk in the future [1]. For example, climate change-induced under-nutrition will mainly occur in currently food-insecure areas [1]. In the UK, climate change will directly influence health outcomes through changing exposure to heat and cold, air pollution, pollen, food safety risks, disruptions to access to and functioning of health services and facilities, emerging infections and flooding, and indirectly via changing prices of and access to food and energy [2].

Health impacts of climate change will not be the same for all because of the differential exposure, sensitivity and adaptive capacity of individuals and groups, which together constitute their vulnerability [3]. For example, people living in flood plains or in top floor flats can be disproportionately exposed to climate change impacts that can affect their health. Older people and people with medical conditions can be disproportionately sensitive to climate change impacts. Finally, isolated people, those with limited mobility and immigrants with limited language skills and local knowledge may have limited adaptive capacity. The nature of health outcomes an individual or a group experiences is determined by the way in which their exposure, sensitivity and adaptive capacity construe their vulnerability: when they are aligned vulnerability is compounded.

Comment [GW1]: Not sure this is the right word … constitute would be better

In what follows, the evidence reported by the other articles of this special issue is interpreted in light of additional literature and social justice reasoning to draw out the key implications of health and social inequalities for the health outcomes of climate change and *vice versa*.

Review

Exposure to heat and cold, air pollution, pollen, food safety risks, disruptions to access to and functioning of health services and facilities, emerging infections and flooding will be examined below, to draw out how health and social inequalities play into the observed and predicted health outcomes of climate change impacts.

Hotter summers and heat waves

Climate change is expected to increase temperature by 2-5°C and increase the frequency and intensity of heat waves in the UK by 2100. Research has established a link between increased mortality and increased temperature and heat waves. Heat related excess deaths occur primarily as a result of respiratory and cardiovascular illnesses [4]. In England and Wales, mortality increases 2.1% for each 1°C increase in temperature above the 93rd percentile of average yearly temperature. The excess deaths caused by increased temperature can include substantial mortality displacement or "harvesting", while it plays a much smaller role in mortality due to heat waves. Heat related mortality is projected to increase in the UK by 45 % by 2020s and by 167% by 2050s [5].

Exposure to heat is greater in the South and East of the country, and people living in urban settlements are more exposed than those living in rural areas due to the urban heat island (UHI) effect. Densely built neighbourhoods with limited open space and green areas increase people's exposure to heat, but the geometry of the buildings and how they are built also influence exposure [6]. Top floor flats experience great thermal stress while ground floor flats do not. Ventilation has substantial influence on heat exposure – it may be constrained by physical building design or by crime and safety considerations. Many of these factors leading to greater exposure come together in deprived urban neighbourhoods.

Older people are more sensitive to heat because of their weaker ability to thermo-regulate [6], and because they have other medical conditions. They are also more likely to have prescribed medication, some of which is associated with increased risk for heat related death. Their adaptive capacity may be limited by isolation or lack of information, mobility or autonomy. Lack of autonomy and lack of care staff awareness and preparedness may, for example, ~~prevent~~ obstruct behavioural and other adaptations in residential or nursing homes. Alignment of the above factors accentuates vulnerability.

Comment [GW2]: This is important as well, combined with the lack of autonomy and/or capacity of older people at risk

Comment [GW3]: For a reference to support this see recent JRF report on 'care provision fit for a future climate'

Milder winters

Cold weather causes thousands of excess deaths in the UK annually. Many of them are caused by cardio-vascular and respiratory illnesses, which are aggravated by cold spells [7]. Cold-related excess deaths do not mainly occur in already frail individuals near the end of their lives unlike is the case with heat-related deaths [7]. Milder winters are predicted to reduce winter mortality and morbidity by 9% by 2020s and 26% by 2050s [5; cf. 8].

Comment [GW4]: This surprises me, is it the conclusion of multiple studies, and generally understood to be the case? Ok if so. Certainly as stated below exposure to the cold exacerbates existing health conditions.. Might want to reference the PHE report that underpins their recent shift in guidance on 'safe' indoor room temperatures

Fuel poverty and poor housing elevate exposure to cold-related deaths [7]. Older people often suffer from both and can thus be more exposed than other groups. Those living in council housing may be less exposed than those living in their own property, as income limitations may prevent older people from improving thermal comfort of their home. They are also more sensitive to cold because they cannot thermo-regulate their bodies as well as other age groups [7]. Sensitivity to cold is increased by pre-existing medical conditions such as cardiovascular or respiratory diseases, which are more prevalent in older age groups. Limited economic and informational resources in turn may weaken the adaptive capacity of older people.

Comment [GW5]: But those privately renting can be in the worst situation ..

A warmer climate may thus generate health benefits by reducing mortality and morbidity among the vulnerable. But while milder winters can reduce cold-related deaths, their

causation is not well understood [9]. Also, demographic change will make a larger number of people sensitive to cold than today [10]. Increasing energy prices may make it more difficult for exposed and sensitive people to adapt by increasing indoor temperature. There is also uncertainty regarding how higher annual and winter average temperatures will be achieved: they may be consistent with the continued occurrence of short cold spells in the future.

Air pollution

The pollution of air by nitrogen dioxide (NO_2), ozone (O_3) and particulate matter (PM) is associated with increased all-cause and cardiovascular mortality and morbidity, and exposure to ozone is associated with increased respiratory mortality and morbidity [11]. Warmer weather, more frequent heat waves, changes in rainfall and altered volatile organic compound concentrations may increase future O_3 and PM concentrations, although their or their pre-cursors' emissions could decrease over time [11]. The evidence from recent heat waves such as that of 2003 suggests that in the UK a third of the excess mortality experienced during a heat wave may be caused by exposure to elevated concentrations of O_3 and PM_{10} [11].

CO and NO_2 concentrations are particularly high in cities near major transport corridors where ethnic minorities are over-represented [12]. If PM concentrations have similar distribution patterns then their increase in changing climate would increase the exposure of the same people to the adverse health outcomes of PM. The situation with O_3 is more complex as high urban NOX concentrations inhibit the formation of O_3. However, high O_3 concentrations can prevail in future if public policies will bring down NOX emissions [13].

Comment [GW6]: Also associated with deprivation/poverty as shown in multiple studies…is it clear that there is an additional ethnic minority over-representation?? And the study referenced is for birmingham only – not clear that the finding applies to other urban areas. The general Uk-wide association with deprivation – in particular exceedences of standards is much better established e.g. see Gordon mitchell's work. Better therefore to emphasise this?

Social deprivation and age pre-dispose people for cardiovascular illnesses and make them sensitive to elevated O_3 and PM concentrations. In 2008, the most deprived quintile in the UK experienced 50% higher cardiovascular disease mortality than the least deprived quintile

[14]. Women in routine jobs experience five times higher cardiovascular disease mortality than women in managerial and professional jobs. These differences in cardiovascular mortality risk and sensitivity to O_3 and PM pollution emerge from differences in the levels of deprivation, lifestyles, health literacy, access to health services, and environmental exposure [15]. Social deprivation and ethnicity can also constrain adaptive capacity by limiting ability to relocate and to take other measures to avoid exposure or to reduce sensitivity.

Pollen and human health

Pollen is one key factor in asthma, which can trigger inflammation of airways, coughing and breathing difficulties among people whose immune systems have become hypersensitive to triggers like pollen [16]. Birch and related trees are a key source of pollen in the early pollen season, replaced by grasses later in the season: warming will prolong pollen seasons and can lead to appearance of new sources of pollen when plant species mix changes due to warming. High pollen levels occur during calm and warm high-pressure systems, and rain and westerly winds lower pollen levels because of wet deposition. Events such as thunderstorms can transform pollen into biological aerosols containing allergens and leading to asthma outbreaks. Therefore, global warming and associated increased frequency and intensity of extreme weather events can aggravate the human health impacts of pollen [16].

While exposure to pollen may be greater in rural areas, trees and weeds lead to pollen exposure in urban areas as well. Asthma has nearly 10% prevalence in the UK population, but among 6-7 year olds its prevalence is over 20% [16]. Asthma is more common among lower socio-economic status people and among Afro-Caribbeans but the role of pollen in the incidence of asthma is not well understood [16]. While the young are more exposed to asthma, older people are much more sensitive: a study of asthma mortality in the United States between 2006-2008 indicates that older people with asthma have 5 times higher overall

mortality risk than younger people, and that they also experienced other more adverse outcomes due to acute asthma [17]. Other medical conditions such as cardiovascular disease are common co-morbidities of asthma in ~~the~~ older people [18].

Food borne diseases

Prevalence and impacts of food safety related pathogens campylobacter and salmonella will be affected by climate change. Campylobacter cases have increased and in 2006 they led to 18 000 hospitalisations, 80 deaths and economic losses of £600 million per annum in England and Wales [19]. However, the projections for future campylobacter cases are uncertain because different drivers influence cases occurring via dietary and environmental pathways. Salmonella cases have decreased: 9000-10000 salmonella cases have been reported to the Health Protection Agency in recent years. These have led to 8500 hospital admissions, 199 deaths and economic losses of £39 million per annum in England and Wales [19]. Climate change may countervail the reduction in salmonella cases brought about by improved food hygiene over the past two decades.

In terms of exposure, campylobacter is more prevalent in rural areas and in areas with less social deprivation [19-20]. It is also more common in infants than in adults and older age groups. Poultry is the most common dietary source of campylobacter. The geographic prevalence of salmonella has not been studied in the UK. Salmonella is typically contracted from raw or undercooked eggs or poultry and it is most common in small children. The elderly and households with small children are the two groups for whom food safety will be a particular problem because of the greater sensitivity of the infants and elderly. Their situation can be exacerbated by low incomes, isolation and other factors that reduce adaptive capacity.

Health and social care systems

Climate change will affect future health outcomes directly through extreme weather events such as heat waves, cold spells, and flooding impacting on the built infrastructure and social and institutional systems of health care provision, and indirectly due to induced changes in the volume and structure of demand for health care [21]. Heat waves and cold spells can put systems of health care under pressure because of the mortality and morbidity increase for cardiovascular and respiratory diseases and associated increases in hospitalisations [21]. Floods in turn result in increased accidents and emergency visits. Climatic extremes can increase ambulance call out rates by 25-35 %, which is comparable to increases related to major flu epidemics [22]. They also increase mental health problems and demand for services addressing them, and influence the length of time that support addressing them is needed [22]. Direct impacts of extreme weather on health care systems include heat stress on inpatients and adverse health outcomes associated with heat stress discussed above, potential care and service disruptions because of power outages, delays in emergency responses and reduced access to health care because of the impacts of flooding and extreme weather on transport infrastructure and services, and reduced staffing and capacity for the same reasons in health care provision [21].

Inpatients and people with urgent medical needs will be most exposed to the impacts of extreme weather on health care systems. Rural dwellers are more exposed to disturbance from cold spells and flooding, while urban residents are more exposed to disturbance due to heat waves. Many of the exposed will be older people who are sensitive to care disruptions and reduced access to care because of pre-existing medical conditions such as cardio-vascular disease and respiratory diseases, which are aggravated by climate change impacts. Adaptive

capacity is restricted among those who are in residential care and have limited control over their circumstances, and among those who are isolated or have reduced mobility.

Emerging infections

Emerging infections are newly appearing in a population or rapidly increasing in incidence or expanding in range, for reasons of which climate change is only one [23]. Climate change can lead to emerging infections when it influences pathogens causing a disease: this is most likely when pathogens spend part of their lives outside the host, making vector-borne diseases the most important infections influenced by climate change [24]. For example, climate change is expected to influence the distribution of malaria, West Nile virus, Chikungunya fever, dengue, Leishmaniasis, Lyme's disease and tick-borne encephalitis (TBE).

Health and social inequalities influence the outcomes of emerging infections in many ways. Factors influencing and patterns of vector ranges can lead to North-South, urban-rural and other gradients in exposure. Differential exposure can align with income or/and age disparities associated with differences in sensitivities and adaptive capacities. For example, if older rural population is more exposed Lyme's disease and TBE, the people at greater risk of these diseases would also be more likely to have other pre-existing medical conditions and thus likely to experience more adverse health outcomes because of the emerging infections. People in rural areas may have lower adaptive capacity because of more limited access to health care services and medical expertise on emerging infections.

Comment [GW7]: Are these well understood and evidenced? If not would be worth saying so

Comment [GW8]: No references in this paragraph? Links to previous comment

Flooding

Climate change is likely to increase the frequency and intensity of surface, riverine and coastal flooding in the UK [25]. Every sixth property (5.2 million properties) is exposed to some flood risk in the UK [26]. Figures for the number of people exposed to flooding in the UK range from 1.5 million to 5 million [27-28]. Damage caused by flooding to buildings and

contents of residential and non-residential properties amounts to about £1.3 billion per annum on average and in years of severe flooding such as 2007 losses can double. The above figures exclude loss of income due to disturbance caused by flooding and additional expenses to make alternative arrangements for leisure, work or business. These indirect losses can add 25-50% to direct losses to buildings and contents [28-29].

Direct health impacts of flooding include drowning, electrocution and other accidental deaths and injuries. Indirect health impacts can occur due to contamination and loss of water supply and loss of access to transport, electricity supply and communications [30-32]. Economic losses and disturbance can also lead to mental health problems [33]: people flooded by the 2007 summer floods in Gloucestershire and Yorkshire were 2-3 times more likely than non-affected people to report mental health problems [34; see also 35-36). Flooding can also undermine health care provision by overwhelming the capacity of emergency services, causing power cuts and supply disruptions, and flooding health care facilities [32, 37].

The risk of flooding and its adverse health impacts are unevenly distributed in several ways. Those living in affordable housing and socio-economically disadvantaged households are over-represented in areas at risk from coastal flooding, while affluent households are more exposed to riverine flooding [38]. Second, people with disabilities, chronic illness, young children or dependent on public transport can be more sensitive to flood risk. Finally, the capacity of some groups to adapt or recover can be lower because of low incomes or lack of insurance: uninsured losses to assets cannot be recovered and repeated exposure to flooding can deplete vulnerable households' assets, adversely affect their health and make them more vulnerable in the future [36, 39]. People's tenure status and type of dwelling may also influence the outcomes they experience.

Comment [GW9]: But there can be important regional and local variation ... in some places there are major concentrations of deprived populations at risk from river flooding

Discussion

The previous section examined how climate change is likely to have differential health impacts on different groups of people, because of differences in their exposure, sensitivity and adaptive capacity. Old age, having pre-existing medical conditions and social deprivation are key attributes that make some people more vulnerable than others to the health impacts of climate change and to experience more adverse health outcomes than others. However, other attributes such as living in rural or urban locations, isolation, marginalisation and weak community cohesion can also make people more vulnerable.

Climate change, aging population and public spending cuts on health and social care can combine to increase health and social inequalities related to health impacts of climate change in the future. The number of people who are over 60 years old will increase by 50% by 2035 compared to 2010 [40]. The proportion of people who are over 75 will double by 2060 compared to 2010 [41]. This means that many more people will have pre-existing medical conditions that make them vulnerable to health impacts of climate change when the impacts of changing climate accentuate. The Lancet Commission estimates that demographic change accounts for three quarters of the increase in the number of people exposed to heat waves and that climatic change only accounts for one quarter of them [9]. At the same time, the systems of health care depend on critical infrastructure for the supply of electricity and water and for communication – this critical infrastructure is exposed and sensitive to the impacts of climate change, which can undermine care [37].

But ageing will also – together with income increase, technological change and increase in the cost of health care provision – create pressure to increase spending on health care from

the current about 8% of GDP to 12-14% of GDP by 2040 [41]. This can be addressed by increasing public spending, reallocation of public spending from other areas to health care, or rationing or patient co-funding of health care provision. If health care is rationed or co-funded, those with greater needs and limited means will suffer reduced access to health care, which will accentuate health and social inequalities.

Many strategies can address health impacts of climate change and the implications of health and social inequalities for them. These include for example new food safety and building regulations, incentivising the refurbishment of old building stock and urban neighbourhoods, improvements to the operations and infrastructure of emergency services and health care provision, improved advance warning and preparedness systems, development and deployment of new diagnostic and therapeutic solutions and public health campaigns and health education. None of these solutions will suffice on its own because compound inequalities underpin vulnerability [42]: a combination of measures will be needed.

Individuals and organisations have capacity for autonomous adaptation and one strategy is to harness it. The strategy of private preparedness and responsibility would emphasise public information campaigns and public health education to make people aware of the health risks associated with climate change, factors contributing to people's exposure and sensitivity, and alternatives for avoiding and mitigating adverse health outcomes, so that they can protect themselves. Solutions such as advance warning systems would also support such strategies, signalling that people should deploy avoidance measures.

But strategies relying on people's own initiative can increase rather than decrease health and social inequalities. Income and wealth inequalities often expose low-income groups to greater

risks because of their residential and other choices, and their adaptive capacity can also be lower than that of higher-income groups. For example, income and wealth inequalities and different levels of trust and engagement translate to different uptake of avoidance measures among different groups of people. Educational status, immigration status and age may also influence people's ability to translate health education into plans and actions, as well as to undertake avoidance measures. There is also evidence that people may not consider taking action to adapt to climate change to be their responsibility [43-44].

Public preparedness measures on the other hand have good potential for alleviating health impacts of climate change and related health and social inequalities. For example, early warnings for emergency services and public service delivery organisations may confer important benefits to vulnerable groups if they lead to improved preparedness and existence of joined-up contingency and emergency plans in different service delivery organisations [39]. This is clearly the aim of the Heat Wave Plan for England [45]. The emergency services are governed by different legislation and priorities than, for example, flood risk management, which can make them better able to prioritising vulnerable groups [46; 47].

Conclusions

Climate change will exacerbate existing health problems in populations that are already most affected and will reduce their resilience to future threats to their wellbeing. Hotter summers, heat waves, milder winters, pollen, air pollution, flooding, emerging infections and food safety are key climate change-related factors influencing health outcomes in the UK. But climate change can also directly impact systems and facilities of health and social care and have indirect impacts on health via changing prices of food and energy.

Climate change will have differential health impacts on different groups of people, because of differences in their exposure, sensitivity and adaptive capacity. Age, pre-existing medical conditions and social deprivation are the key factors that make people vulnerable to health impacts of climate change and to experience more adverse health outcomes than others. But living in rural or urban places, isolation, marginalisation and social embeddedness in and cohesiveness of communities also influence vulnerability. Climate change, aging population, decreasing public spending on health and social care can combine to increase in the future health and social inequalities to do with climate variability and change.

While existing research on climate change impacts and health sheds some light on the implications of health and social inequalities for health outcomes of climate change impacts, less is known about the implications of adaptation responses to address them. Different strategies for avoiding and mitigating health impacts of climate change can have quite different implications for health and social inequalities. For example, health education and public preparedness measures that take into account differential exposure, sensitivity and adaptive capacity of different groups may help to address health and social inequalities. Strategies based on individual preparedness, action and behaviour change may in turn aggravate them due to selective uptake of measures and the lack of engagement of some social groups, unless coupled with broad public information campaigns. More research is needed on the implications of response strategies.

Competing interests

The author declares no competing interest to the authorship and/or publication of this article

Authors' contributions

The sole author undertook all of the design, review and analysis reported in the article

Authors' information

[1] Centre for Climate Change Economics and Policy (CCCEP), School of Earth and Environment, University of Leeds, Leeds, UK

Acknowledgements

Jouni Paavola acknowledges financial support from the Living with Environmental Change (LWEC) programme for the writing of an earlier version of this article. The article also contributes to the research programme of the Centre for Climate Change Economics and Policy (CCCEP), a joint research centre of University of Leeds and London School of Economics and Political Science. The financial support of the UK Economic and Social Research Council (ESRC) to CCCEP is gratefully acknowledged.

References

1. Climate Change 2014: Impacts, Adaptation and Vulnerability. Working Group II Contribution to the Fifth Assessment Report. Intergovernmental Panel on Climate Change (IPCC), 2014.

2. Morris, GP, Reis S, Beck S, Fleming LE, Adger WN, Benton TG, Depledge MH: Climate change and health in the UK: scoping and communicating the long-term "distal" dimensions. *Environ Health*, in review.

3. Adger WN: Vulnerability. *Global Environ Chang.* 2006; 16: 268-81.

4. Arbuthnott K, Hajat S: Hotter summers and heat waves. *Environ Health*, in review.

5. Hajat S, Vardoulakis S, Heaviside C, Eggen B: Climate change effects on human health: projections of temperature-related mortality for the UK during the 2020s, 2050s and 2080s. *Journal of Epidemiology and Community Health*, 2014. doi:10.1136/jech-2013-202449.

6. Oikonomou E, Davies M, Mavrogianni A, Biddulph P, Wilkinson P, Kolokotroni M: Modelling the relative importance of the urban heat island and the thermal quality of dwellings for overheating in London. *Build Environ.* 2012; 57: 223-38.

7. Hajat S: Health effects of milder winters. *Environ Health*, in review.

8. Staddon PL, Montgomery HE, Depledge MH: Climate warming will not decrease winter mortality. *Nature Climate Change.* 2012; 4: 190-94.

9. Watts N, Adger WN, Agnolucci P, et al: Health and climate change: policy responses to protect public health. *Lancet*. 2015.

10. Oven KJ, Curtis SE, Reaney S, et al: Climate change and health and social care: Defining future hazard, vulnerability and risk for infrastructure systems supporting older people's health care in England. *Appl Geogr*. 2012; 33: 16-24.

11. Doherty R, Heal M, O'Connor F: Climate change impacts on human health through its effects on air quality. *Environ Health*, in review.

12. Brainard, JS, et al: Modelling environmental equity: access to air quality in Birmingham,

England. *Environ Plann A*. 2002; 34: 695–716.

13. Heal MR, Heaviside C, Doherty RM, Vieno M, Stevenson DS, Vardoulakis S: Health burdens of surface ozone in the UK for a range of future scenarios. *Environ Int*. 2013; 61: 36–44.

14. Bridging the gaps: tackling inequalities in cardiovascular disease. Heart UK, 2013. http://heartuk.org.uk/files/uploads/Bridging_the_Gaps_Tackling_inequalities_in_cardiovascular_disease.pdf. Accessed 26 May 2016.

15. The Marmot Review. Fair Society, Healthy Lives, 2011. http://www.instituteofhealthequity.org/projects/fair-society-healthy-lives-the-marmot-review. Accessed 26 May 2016

16. Osborne NJ, Eggen B: Pollen and human health: impacts of anthropogenic climate change. *Environ Health*, in review.

17. Tsai C-L, Lee W-Y, Hanania NA, Camargo CA: Age-related differences in clinical outcomes for acute asthma in the United States, 2006-2008. *J Allergy Clin Immunol*. 2012; 129: 1252-8.

18. Yáñez et al.: Asthma in the elderly: what we know and what we have yet to know. *World Allergy Organ J*. 2014; 7:8.

19. Lake I: Food borne disease and climate change. *Environ Health*, in review.

20. Nichols GL, Richardson JF, Sheppard SK, Lane C, Sarran C. Campylobacter epidemiology: A descriptive study reviewing 1 million cases in England and Wales between 1989 and 2011. BMJ Open. 2012; 2.

21. Curtis S, Fair A, Wistow J, Val D, Oven J: Impact of extreme weather events and climate change for health and social care systems. *Environ Health*, in review.

22. Thornes JE, Fisher PA, Rayment-Bishop T. Ambulance call-outs and response times in Birmingham and the impact of extreme weather and climate change. *Emergen Med Journal*. 2014; 31(3):220-28. doi:10.1136/emermed-2012-201817.

23. Morens DM, Folkers GK, Fauci AS: The challenge of emerging and re-emerging infectious diseases. *Nature*. 2004; 430: 242-249.

24. Baylis M: Potential impact of climate change on emerging infections in the UK. *Environ Health*, in review.

25. The UK Climate Change Risk Assessment 2012: Evidence Report. Department of Environment, Food and Rural Affairs (DEFRA), 2012. http://randd.defra.gov.uk/Default.aspx?Module=More&Location=None&ProjectID=15747. Accessed 26 May 2016.

26. Flooding in England: A National Assessment of Flood Risk. Environment Agency, 2009. https://www.gov.uk/government/uploads/system/uploads/attachment_data/file/292928/geho0609bqds-e-e.pdf. Accessed 26 May 2016.

27. Lindley S, O'Neill J, Kandeh J, Lawson N, Christian R, O'Neill M. Climate Change, Justice and Vulnerability. Joseph Rowntree Foundation, 2011. http://www.jrf.org.uk/publications/climate-change-justice-and-vulnerability. Accessed 26 May 2016.

28. Watkiss P, Hunt A. Scoping study: reviewing the coverage of economic impacts in the CCRA. Committee on Climate Change, Adaptation Sub-Committee, 2012. http://www.paulwatkiss.co.uk/newimagesanddocs/Filling%20the%20Gaps%20around%20the%20CCRA%20Working%20Paper%20vs%208.pdf. Accessed 26 May 2016.

29. Lantz V, Trenholm R, Wilson J, Richards W: Assessing market and non-market costs of freshwater flooding due to climate change in the community of Fredericton, Eastern Canada. *Climatic Change* 2012; 110: 347–372.

30. Milojevic, A: Flooding and health. *Environ Health*, in review.

31. The effects of flooding on mental health. Health Protection Agency, 2011. http://webarchive.nationalarchives.gov.uk/20140714084352/http:/www.hpa.org.uk/webc/HPAwebFile/HPAweb_C/1317131767423. Accessed 26 May 2016.

32. Floods in the WHO European Region: health effects and their prevention. WHO, 2013. http://www.euro.who.int/__data/assets/pdf_file/0020/189020/e96853.pdf. Accessed 26 May 2016.

33. Stanke C, Murray V, Amlôt R, Nurse J, Williams R: The effects of flooding on mental health: outcomes and recommendations from a review of the literature. Currents Disasters. 2012. Edition 1. doi: 10.1371/4f9f1fa9c3cae.

34. Paranjothy S, Gallacher J, Amlot R, Rubin GJ, Page L, Baxter T, etal. Psychosocial impact of the summer 2007 flood in England. *BMC Public Health*. 2011; 11: 145.

35. Mason V, Andrews H, Upton D: The psychological impact of exposure to floods. *Psychology Health & Medicine*. 2010; 15**:** 61-73.

36. Whittle R, Medd W, Deeming H, Kashefi E, Mort M, Twigger Ross C, Walker G, Watson N. After the Rain – learning the lessons from flood recovery in Hull. Lancaster University, 2010. http://www.lancaster.ac.uk/lec/sites/cswm/Hull%20Floods%20Project/AFTER%20THE%20RAIN%20FINAL%20REPORT.pdf. Accessed 26 May 2016.

37. Klinger C, Landeg O, Murray V. Power Outages, Extreme Events and Health: a Systematic Review of the Literature from 2011-2012. *Current Disasters*. 2014; Jan 2. Edition 1. doi 10.1371/currents.dis.04eb1dc5e73dd1377e05a10e9edde673.

38. Houston D, Werritty A, Bassett D, Geddes A, Hoolachan A, McMillan M. Pluvial (rain-related) flooding in urban areas: the invisible hazard. Joseph Rowntree Foundation, 2011. http://www.jrf.org.uk/publications/pluvial-flooding-invisible-hazard. Accessed 26 May 2016.

39. Pringle P, Paavola J, Dale N, Sibille R, Kent N, Le Cornu E. *PREPARE:* Understanding equity and distributional impacts of climate risks and adaptation options. Ricardo -AEA, 2013. http://www.ricardo-aea.com/cms/assets/Documents/11257PREPARECA0513Socialvulnerability-Finalreport.pdf. Accessed 26 May 2016.

40. 2010-based National Population Projections Reference Volume: Chapter 2: Results. Series PP2. Office for National Statistics, 2012. http://www.ons.gov.uk/ons/rel/npp/national-population-projections/2010-based-reference-volume--series-pp2/results.html. Accessed 26 May 2016.

41. Appleby J. Spending on health and social care over the next 50 years: Why think long term. The King's Fund, 2013. http://www.kingsfund.org.uk/sites/files/kf/field/field_publication_file/Spending%20on%20health%20…%2050%20years%20low%20res%20for%20web.pdf. Accessed 26 May 2016.

42. Preston I, Banks N, Hargreaves K, Kazmierczak A, Lucas K, Mayne R, Downing C, Street R. Climate change and social justice: an evidence review. Joseph Rowntree Foundation, 2014. http://www.jrf.org.uk/sites/files/jrf/climate-change-social-justice-full.pdf. Accessed 26 May 2016.

43. Bichard E, Kazmierczak A: Are homeowners willing to adapt to and mitigate the effects of climate change? *Climatic Change.* 2012; 112: 633-54.

44. Williams K, Gupta R, Smith I, Joynt J, Hopkins D, Bramley G, Payne C, Gregg M, Hambleton R, Bates-Brkljac N, Dunse N, Musslewhite C. Suburban Neighbourhood Adaptation for a Changing Climate (SNACC) Final Report, 2012. https://snacc.files.wordpress.com/2012/10/snacc_report.pdf. Accessed 26 May 2016.

45. Heatwave plan for England: protecting health and reducing harm form severe heat and heatwaves. Public Health England, 2015. https://www.gov.uk/government/uploads/system/uploads/attachment_data/file/429384/Heatwave_Main_Plan_2015.pdf. Accessed 26 May 2016.

46. Johnson C, Penning-Rowsell E, Parker D: Natural and imposed injustices: the challenges in implementing 'fair' flood risk management policy in England. *Geogr J.* 2007; 173: 374–90.

47. Brisley R, Welstead J, Hindle R, Paavola J. Socially just adaptation to climate change. Joseph Rowntree Foundation, 2012. http://www.jrf.org.uk/sites/files/jrf/climate-change-adaptation-full_0.pdf. Accessed 26 May 2016.

Author response

The first reviewer (Vardoulakis) provided the most detailed comments, highlighting ambiguities in my argumentation and insufficient referencing, as well as suggesting additional sources to consult. I have addressed all of these more detailed comments. These revisions should also help to address the second reviewer's minor revision comment (p. 2 on the report) on the need for more citations to support specific assertions.

Both the first and second reviewer in their own ways identify a weakness in the positioning of the manuscript / in rationalising what it does. Refer to the review paragraph of the first reviewer and the "summary and proposed revisions" part of the report of the second referee.

They are quite right – the paper was commissioned as a social justice re-interpretation of the results of the other contributions to the special issue, in their earlier incarnations as technical papers underpinning the climate change report card on health impacts of climate change. When transforming my paper into an article manuscript I clearly had failed to rationalise well enough the need and rationale for the manuscript *as a free-standing journal article.*

I have revised the text of the introduction and the first paragraph of the review section to address the above comments. I have not extended the text substantially because my manuscript is already long considering the author guideline, but I have better set the manuscript to its academic context and explained why and how it makes a contribution. I have also tried to better explain the nature of the exercise undertaken in the paper.

Reviewer 3 provided relatively small number of comments on the manuscript that are similar in nature than the more detailed comments of reviewer 3. They were all well taken and I have incorporated the suggestions and comments to the revised version.

Finally, in her final comment (referring to p. 14) reviewer 2 wonders whether my arguments are consistent. They are, because I am talking about the importance of "public preparedness" – of public service delivery organisations to dispatch their duties. The wording does not appear ambiguous, so I have not made changes in response to this comment, which probably arose from quick reading and association of "preparedness" with private preparedness.
